# Noncalcified plaque burden quantified from coronary computed tomography angiography improves prediction of side branch occlusion after main vessel stenting in bifurcation lesions: results from the CT-PRECISION registry

**DOI:** 10.1007/s00392-020-01658-1

**Published:** 2020-05-08

**Authors:** Kajetan Grodecki, Sebastien Cadet, Adam D. Staruch, Anna M. Michalowska, Cezary Kepka, Rafal Wolny, Jerzy Pregowski, Mariusz Kruk, Mariusz Debski, Artur Debski, Ilona Michalowska, Piotr J. Slomka, Adam Witkowski, Damini Dey, Maksymilian P. Opolski

**Affiliations:** 1 Department of Interventional Cardiology and Angiology, National Institute of Cardiology, Alpejska 42, 04-628 Warsaw, Poland; 2 Medical University of Warsaw, Warsaw, Poland; 3 Biomedical Imaging Research Institute and Artificial Intelligence in Medicine Program, Departments of Biomedical Sciences and Medicine Cedars-Sinai Medical Center, Los Angeles, CA USA; 4 Department of Coronary and Structural Heart Diseases, National Institute of Cardiology, Warsaw, Poland; 5 Department of Radiology, National Institute of Cardiology, Warsaw, Poland

**Keywords:** Coronary computed tomography angiography, Coronary artery disease, Coronary bifurcation, Percutaneous coronary intervention, Plaque burden

## Abstract

**Objectives:**

To assess the incremental value of quantitative plaque features measured from computed tomography angiography (CTA) for predicting side branch (SB) occlusion in coronary bifurcation intervention.

**Methods:**

We included 340 patients with 377 bifurcation lesions in the post hoc analysis of the CT-PRECISION registry. Each bifurcation was divided into three segments: the proximal main vessel (MV), the distal MV, and the SB. Segments with evidence of coronary plaque were analyzed using semi-automated software allowing for quantitative analysis of coronary plaque morphology and stenosis. Coronary plaque measurements included calcified and noncalcified plaque volumes, and corresponding burdens (respective plaque volumes × 100%/vessel volume), remodeling index, and stenosis.

**Results:**

SB occlusion occurred in 28 of 377 bifurcation lesions (7.5%). The presence of visually identified plaque in the SB segment, but not in the proximal and distal MV segments, was the only qualitative parameter that predicted SB occlusion with an area under the curve (AUC) of 0.792. Among quantitative plaque parameters calculated for the SB segment, the addition of noncalcified plaque burden (AUC 0.840, *p* = 0.003) and low-density plaque burden (AUC 0.836, *p* = 0.012) yielded significant improvements in predicting SB occlusion. Using receiver operating characteristic curve analysis, optimal cut-offs for noncalcified plaque burden and low-density plaque burden were > 33.6% (86% sensitivity and 78% specificity) and > 0.9% (89% sensitivity and 73% specificity), respectively.

**Conclusions:**

CTA-derived noncalcified plaque burden, when added to the visually identified SB plaque, significantly improves the prediction of SB occlusion in coronary bifurcation intervention.

**Trial registration:**

ClinicalTrials.gov Identifier: NCT03709836 registered on October 17, 2018.

## Introduction

Treatment of coronary bifurcation lesions accounts for 15–20% of all percutaneous coronary intervention (PCI) procedures [1]. The provisional approach with stenting of the main vessel (MV) first, considered as the preferred strategy in most coronary bifurcation lesions, is associated with an incidence of side branch (SB) occlusion of approximately 10% (a complication that might have serious consequences including clinically significant myocardial infarction and adverse clinical outcomes) [2–5]. Although studies utilizing intravascular imaging techniques identified plaque and carina shift as two major mechanisms of SB compromise, data on the role of plaque morphology in this complication are scarce [6–8].

Recently, non-invasive coronary computed tomography angiography (coronary CTA) has gained recognition as the first-line diagnostic test in subjects with suspected coronary artery disease [9, 10]. As a consequence, the number of patients undergoing CTA before invasive coronary angiography is expected to rise, underscoring the importance of utilizing all computed tomography (CT) information for planning and guiding PCI [11]. Unlike invasive angiography, coronary CTA enables visualization and quantification of atherosclerotic plaque with respect to coronary lumen and vessel geometry [12–15]. Whereas the distribution of coronary plaque included in the CTA-derived RESOLVE score was found to be predictive of SB occlusion, there is paucity of data on the relationship between plaque morphology and the risk of SB compromise in coronary bifurcation interventions [4]. This study, therefore, aimed to assess the incremental value of quantitative morphological plaque features measured from CTA for predicting SB occlusion in coronary bifurcation intervention.

## Methods

### Study design and population

From January 2010 to July 2018, 15,918 consecutive patients underwent PCI at a single high-volume center. Inclusion criteria were: (1) PCI of a bifurcation lesion with significant SB; (2) initial stenting of the main branch with a provisional approach to the SB; (3) performance of coronary CTA within 30 days before attempted PCI. Coronary bifurcation lesion was defined as a coronary artery narrowing occurring adjacent to or involving the origin of a significant SB [15]. Significant SB was defined according to the 11th consensus document from the European Bifurcation Club as a branch that the operator would not want to lose in the global context of an individual patient [16]. The primary endpoint was defined as SB occlusion represented by either any decrease in thrombolysis in myocardial infarction (TIMI) flow grade (including TIMI 1–2 flow grade) or the absence of flow in the SB (TIMI 0 flow grade) after MV stenting [15].

**Fig. 1:**
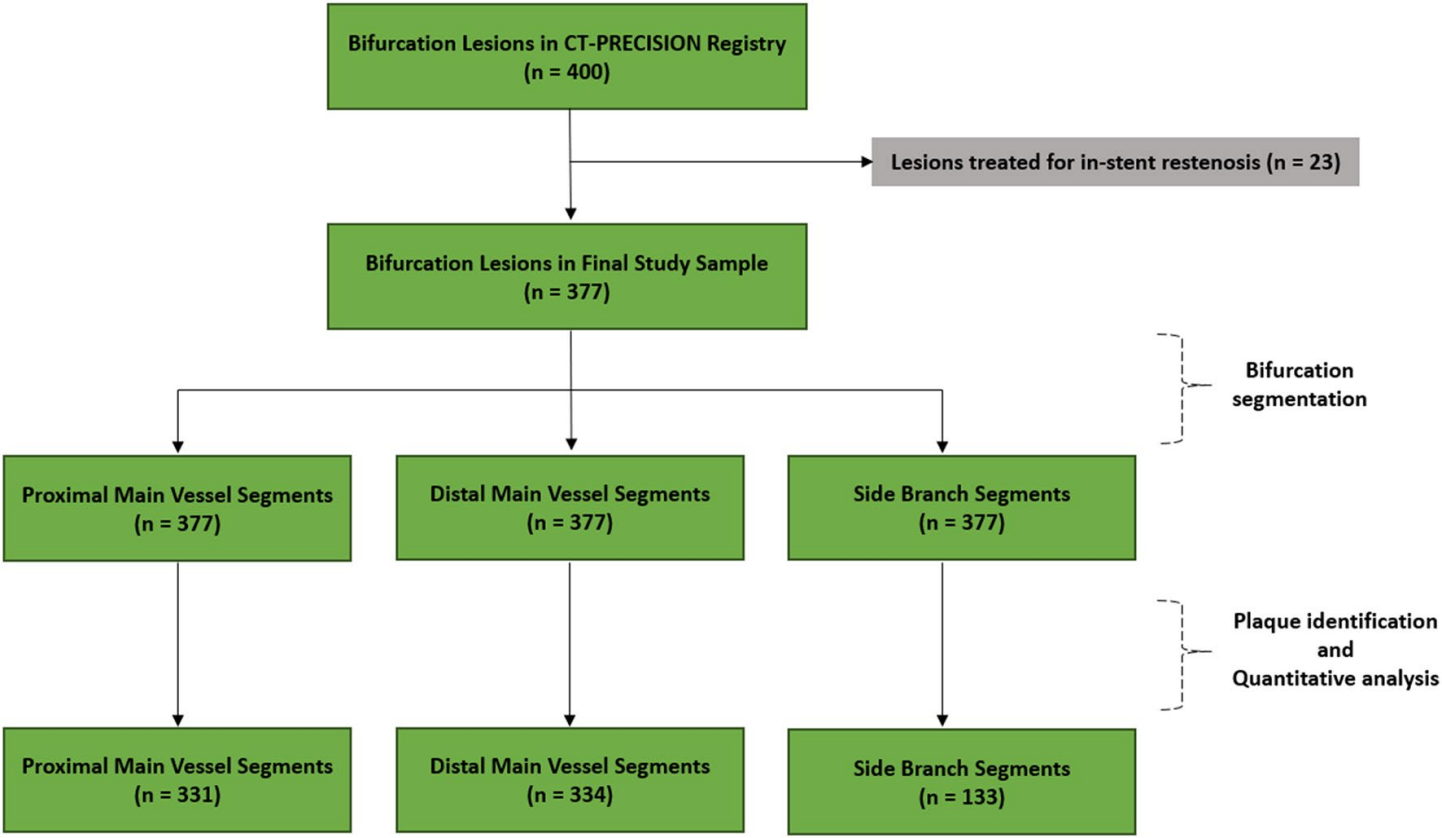
Study flowchart

Our study was a post hoc analysis of the participants in the CT-PRECISION registry (NCT03709836), and was approved by the Institutional Review Board and complied with the declaration of Helsinki. The registry was described previously [4]. Out of 400 bifurcation lesions among 363 patients, we excluded 23 lesions with in-stent restenosis in 23 patients, due to the limitations of quantitative plaque analysis in stented segments. Thus, the final study cohort included 377 bifurcation lesions in 340 patients (Fig. 1).

### CTA protocol and image reconstruction

During the study period, three generations of dual-source CT scanners were used (SOMATOM Definition, SOMATOM Definition Flash and SOMATOM Force, Siemens, Erlangen, Germany). Patients without contraindications received intravenous metoprolol (2.5 mg boluses titrated up to 10 mg) to limit heart rate below 65 beats/min, and sublingual nitroglycerin was administered prior to image acquisition on a regular basis. The contrast transit time was estimated by injection of a test bolus. For acquisition of the volume dataset, 60–120 ml iodinated contrast material (Iomeron 400, Bracco Imaging SpA, Milan, Italy) was injected intravenously at a rate of 6 ml/s (Definition, Flash) or 4.5 ml/s (Force). Injection rate could be decreased as a part of contrast material reduction strategy with lower tube voltages generated by the third-generation dual-source CT scanners (Force). Data were acquired using a retrospectively gated or prospectively electrocardiogram-triggered protocol with a beam collimation of 64 × 0.6 mm (Definition), 128 × 0.6 mm (Flash) or 192 × 0.6 mm (Force) and tube voltage of 70–120 kV adjusted to body mass index. Radiation dose reduction strategies including electrocardiogram-gated tube current modulation and prospectively electrocardiogram-triggered sequential acquisition were used whenever feasible. Image data were reconstructed in mid-to-end systole and diastole (35–45% and 65–75% of the R–R interval) with 0.6-mm slice thickness and 0.4 mm increment.

### CTA analysis

**Fig. 2:**
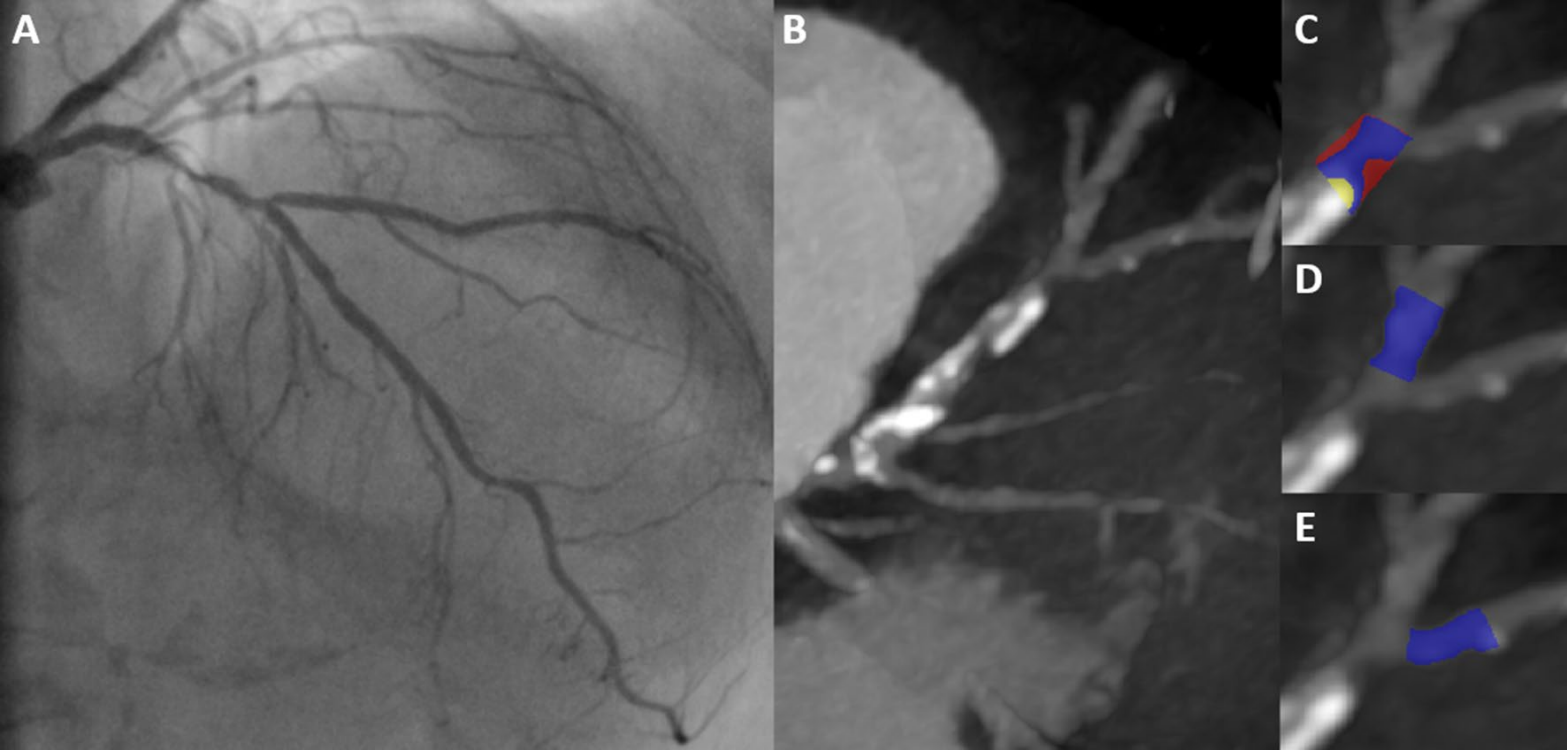
An example of the coronary bifurcation lesion in invasive angiography (**a**) and its corresponding image in computed tomography angiography (**b**). Plaque was quantitatively analyzed in the respective bifurcation segments: proximal main vessel (**c**), distal main vessel (**d**) and side branch (**e**). Blue represents lumen, yellow—calcified plaque, and red—noncalcified plaque. In the above lesion, coronary plaque—defined as tissue greater than 1 mm^2^ associated with the coronary wall—was present only in the proximal segment of the main vessel

Coronary bifurcation was divided into: the proximal MV, the distal MV, and the SB. The beginning of the distal MV segment was defined at the carinal level as previously described [17]. Segments within 5 mm proximal and distal to the carinal point for MV, and 5 mm distal from the ostium of the SB were analyzed [17]. Coronary arteries were assessed with multiplanar, curved multiplanar, and transverse reformations in the diastolic phase of the cardiac cycle. First, each segment of the bifurcation was visually evaluated for the presence of coronary plaque defined as any discernible structure greater than 1 mm^2^ that could be assigned to the coronary artery wall [18]. Segments with evidence of coronary plaque were further analyzed using semi-automated software (AutoPlaque version 2.5, Cedars-Sinai Medical Center, Los Angeles, California) allowing for quantitative 3-dimensional assessments of plaque morphology and stenosis (Fig. 2). As described and validated previously, scan-specific thresholds for calcified and noncalcified plaques were automatically generated. Briefly, the attenuation threshold levels were calculated by fitting a Gaussian curve to the image histogram from the aortic normal blood pool (defined by placing a circular region of interest within the ascending aorta), after adjusting with the proximal-to-distal luminal contrast enhancement distribution in the analyzed vessel [19]. Low-density plaque was defined as noncalcified plaque subset using a fixed attenuation of < 30 Hounsfield units [20]. Coronary plaque measurements included absolute volumes (in mm^3^) and corresponding burdens (respective plaque volumes × 100%/vessel volume). Furthermore, maximal diameter stenosis, maximal area stenosis, minimal lumen diameter, minimal lumen area, vessel remodeling index, and contrast density difference were measured. Maximal diameter stenosis was calculated as the ratio of the narrowest luminal diameter and the mean of two non-diseased reference points within the same bifurcation segment. Maximal area stenosis was calculated as the ratio of the narrowest luminal area and the mean of two non-diseased reference points within the same bifurcation segment [21] Minimal luminal diameter and area were determined semi-quantitatively at the region of maximal stenosis degree [20]. The remodeling index was determined as the ratio between maximum vessel area and the vessel area at the beginning of the bifurcation segment [22]. The luminal contrast density, defined as attenuation per unit area (similar to area gradient), was computed over 1-mm cross-sections of the arterial segment within bifurcation lesion. The contrast density difference was defined as the maximum percent difference in contrast densities across the bifurcation segment with respect to the proximal reference cross-section [23]. True bifurcation lesions were defined as a Medina classification of 0.1.1, 1.0.1, or 1.1.1.

### Statistical analysis

Continuous variables are expressed as the mean ± standard deviation or median and interquartile range, and were compared with Student’s *t*-test or Mann–Whitney *U* test as appropriate. Chi-square or Fisher’s exact tests were used for comparison of categorical variables expressed as counts and percentages. Discriminatory performance was determined by the C-statistic, and compared using the method of DeLong et al. [24] Receiver operating characteristic (ROC) curves were generated for the presence of plaque in respective bifurcation segments alone or in combination with quantitative plaque analysis. In the combined analysis, plaque presence was given hierarchical superiority. Optimal cut-offs for quantitative plaque features were identified using the Youden index. All probability values were two -tailed, and a *p* value of < 0.05 was considered statistically significant. Data were processed using the SPSS software, version 23 (IBM SPSS Statistics, New York, USA).

## Results

### Clinical and procedural characteristics

Out of 377 bifurcation lesions, 1131 bifurcation segments were screened for quantitative plaque analysis (Fig. 1). Overall, the presence of coronary plaque was noted in 331 (87.8%) proximal MV, 334 (88.6%) distal MV, and 133 (35.3%) SB segments. SB occlusion occurred in 28 (7.5%) of 377 bifurcations: the absence of flow and decrease in TIMI flow were noted in 12 (43%) lesions and 16 (57%) lesions, respectively.

The baseline clinical characteristics were similar in patients with and without SB occlusion (Table 1). Per-lesion angiographic characteristics showed higher incidence of true bifurcations in lesions with versus without SB occlusion (Table 2).

**Table 1:** Per-patient baseline characteristics

	No SB occlusion (*n* = 314)	SB occlusion (*n* = 26)	*p* value
Age	64.3 ± 10.0	63.6 ± 11.0	0.724
Male	229 (72.9)	19 (73.1)	0.987
Body mass index (kg/m^2^)	28.0 ± 4.1	28.9 ± 5.1	0.400
Diabetes mellitus	78 (24.8)	6 (23.1)	0.841
Hyperlipidemia	249 (79.3)	21 (80.8)	0.858
Hypertension	250 (79.6)	22 (84.6)	0.888
Smoking history	63 (20.1)	7 (26.9)	0.406
Current smoker	33 (10.5)	3 (11.5)	0.870
Previous myocardial infarction	66 (21.0)	9 (34.6)	0.108
Previous coronary artery bypass grafting	33 (10.5)	3 (11.5)	0.870
Previous PCI	57 (18.2)	8 (30.8)	0.116
Previous stroke	12 (3.8)	0	0.310
Stable coronary artery disease	250 (79.6)	24 (92.3)	0.116
Unstable angina	45 (14.3)	2 (7.7)	0.346
Non-ST-elevation myocardial infarction	15 (4.8)	0	0.254
ST-elevation myocardial infarction	4 (1.3)	0	0.563
Left ventricular ejection fraction	56.8 ± 11.8	54.7 ± 12.6	0.442

Values are presented as *n* (%) or mean ± SD

*PCI* percutaneous coronary intervention, *SB* side branch

**Table 2:** Per-lesion procedural characteristics

	No SB occlusion (*n* = 349)	SB occlusion (*n* = 28)	*p* value
Access site			0.870
Femoral	89 (25.5)	8 (28.6)	
Radial	258 (73.9)	20 (71.4)	
Brachial	2 (0.6)	0	
Location of target lesion			0.211
LM	32 (9.2)	0	
LAD	185 (53.0)	16 (57.1)	
CX	45 (12.9)	2 (7.1)	
RCA	87 (24.9)	10 (35.7)	
Medina classification			0.002
1,0,0	53 (15.2)	2 (7.1)	
0,1,0	56 (16.1)	1 (3.6)	
1,1,0	105 (30.0)	4 (14.3)	
1,1,1	101 (28.9)	17 (60.7)	
0,0,1	0	0	
1,0,1	11 (3.2)	3 (10.7)	
0,1,1	23 (6.6)	1 (3.6)	
Jailed wire in SB	109 (31.2)	5 (17.9)	0.138
Predilation of SB	9 (2.6)	2 (7.1)	0.167
Number of stents			0.603
1	243 (69.7)	21 (75.0)	
2	81 (23.2)	4 (14.3)	
3	20 (5.7)	2 (7.1)	
≥ 4	5 (1.4)	1 (3.6)	
Total stent length (mm)	27.1 ± 15.7	27.1 ± 17.6	0.995

Values are presented as *n* (%) or mean ± SD

*CX* circumflex artery, *LAD* left anterior descending coronary artery, *LM* left main coronary artery, *RCA* right coronary artery, *SB* side branch

### Plaque characteristics associated with side branch occlusion

Table 3 displays quantitative anatomical plaque characteristics stratified by the occurrence of SB occlusion for respective bifurcation segments. While the SB occlusion group displayed higher contrast density difference in the proximal MV segment than the SB non-occlusion group (16.5 vs 9.3%, *p* = 0.034), no significant between-group differences were found in distal MV. Lesions with SB occlusion had lower minimal luminal diameter (1.5 ± 0.6 vs 2.0 ± 0.7 mm, *p* = 0.002) and minimal lumen area (1.9 [IQR 0.9–2.6] vs 3.1 [IQR 1.7–4.6] mm^2^, *p* = 0.003) within SB segments compared to lesions without SB occlusion.

**Table 3:** Quantitaive lesion characteristics for respective bifurcation segments with visually identified coronary plaque

	Proximal main vessel			Distal main vessel			Side branch		
	No SB occlusion (*n* = 304)	SB occlusion (*n* = 27)	*p* value	No SB occlusion (*n* = 306)	SB occlusion (*n* = 28)	*p* value	No SB occlusion (*n* = 108)	SB occlusion (*n* = 25)	*p* value
Diameter stenosis (%)	18.6 (12.9–29.2)	27.2 (12.9–29.2)	0.713	19.6 (11.9–29.7)	18.6 (10.9–33.3)	0.945	18.9 (13.7–29.2)	20.3 (15.4–28.6)	0.582
Area stenosis (%)	34.4 (22.0–52.0)	40.2 (24.4–51.5)	0.748	35.6 (22.9–52.2)	33.9 (21.0–56.4)	0.922	34.9 (26.2–51.0)	37.4 (28.5–49.2)	0.553
Minimal lumen diameter (mm)	2.5 ± 0.9	2.4 ± 0.6	0.492	2.4 ± 0.7	2.4 ± 0.9	0.953	2.0 ± 0.7	1.5 ± 0.6	0.002
Minimal lumen area (mm^2^)	5.1 (3.0–7.7)	5.2 (2.9–6.9)	0.552	4.4 (2.9–6.4)	3.8 (2.1–7.0)	0.668	3.1 (1.7–4.6)	1.9 (0.9–2.6)	0.003
Remodeling index	1.1 (1.0–1.1)	1.1 (1.0–1.1)	0.408	1.0 (1.0–1.1)	1.0 (1.0–1.1)	0.106	1.0 (1.0–1.1)	1.0 (1.0–1.1)	0.880
Contrast density difference (%)	9.3 (1.4–19.4)	16.5 (8.2–31.1)	0.034	13.6 (2.9–23.5)	8.1 (0.7–16.5)	0.097	9.4 (1.0–19.2)	15.2 (− 2.4–32.4)	0.567

Values are presented as mean ± SD or median (interquartile range)

**Fig. 3:**
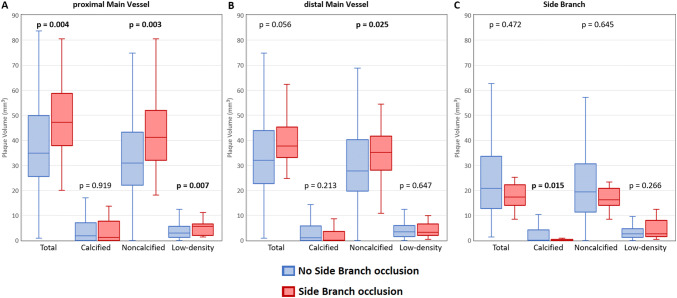
Comparison of plaque volumes in respective bifurcation segments with and without SB occlusion

**Fig. 4:**
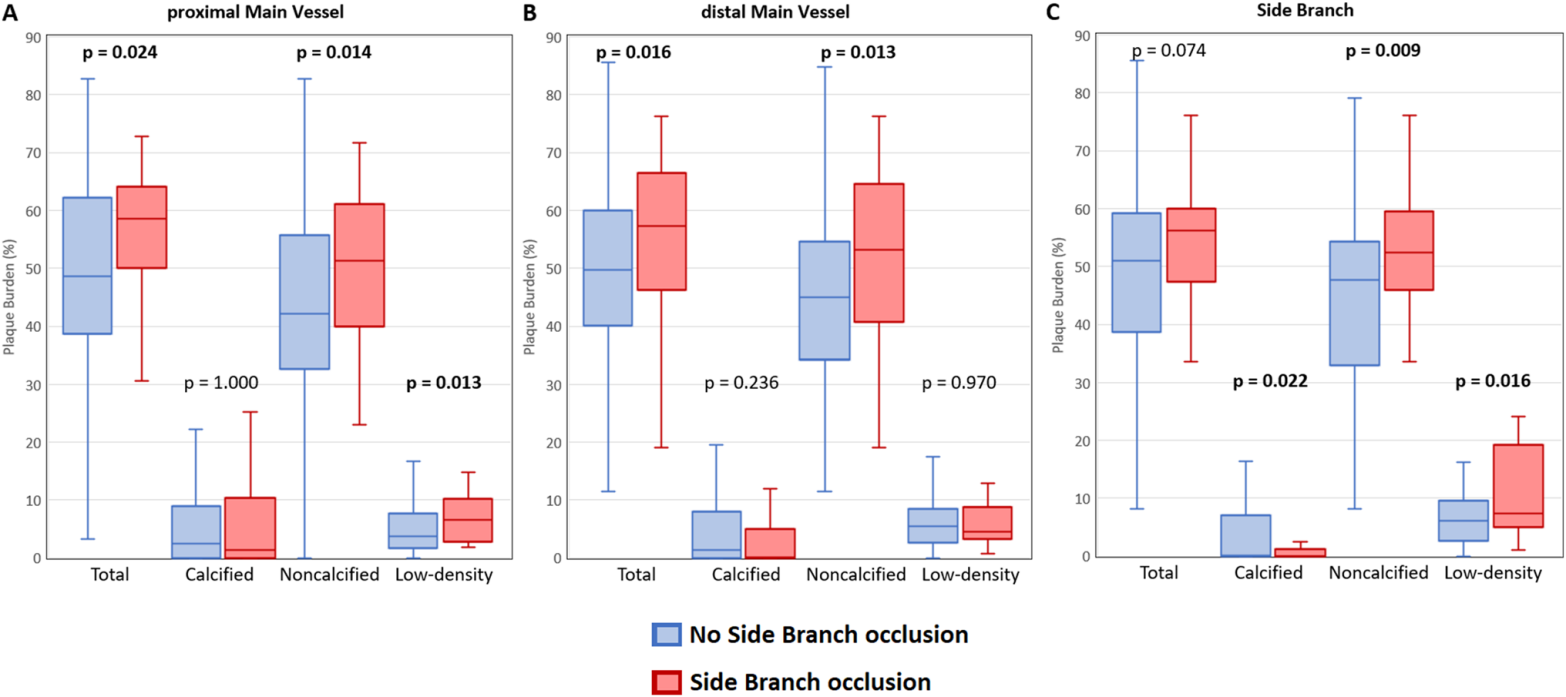
Comparison of plaque burden in respective bifurcation segments with and without SB occlusion

Quantitative morphological plaque characteristics stratified by the presence of SB occlusion are summarized in Figs. 3, 4. Lesions with occluded SB had significantly higher total plaque volume compared to lesions without SB occlusion in proximal MV segment (47.2 [IQR 37.9–58.7] vs 34.9 [IQR 25.5–49.9] mm^3^, *p* = 0.004) (Fig. 3). Whereas SB occlusion group demonstrated higher volumes of noncalcified plaque in proximal (41.2 [IQR 32.0–52.0] vs 31.0 [IQR 22.0–43.3] mm^3^, *p* = 0.003) and distal (35.3 [IQR 28.0–41.6] vs 27.8 [IQR 19.6–40.2] mm^3^, *p* = 0.025) MV segments, the volume of low-density plaque differed only in proximal MV with higher values in the SB group than non-SB group (5.7 [IQR 2.0–6.7] vs 3.0 [IQR 1.2–5.7] mm^3^, *p* = 0.007). Calcified plaque volume differed between the two groups exclusively in the SB segment, where lesser volume was observed in lesions with SB occlusion than without SB occlusion (0.0 [IQR 0.0–0.1] vs 0.0 [IQR 0.0–4.2] mm^3^, *p* = 0.015).

Both proximal (58.75 [IQR 50.0–64.1] vs 48.7 [IQR 38.6–62.3]%, *p* = 0.024) and distal (57.3 [IQR 46.3–66.5] vs 49.7 [IQR 40.1–59.9]%, *p* = 0.016) segments of MV but not SB (56.2 [IQR 47.3–60.0] vs 51.0 [IQR 38.6–59.3]%, *p* = 0.074) had higher total plaque burden in lesions with than without SB occlusion. Whereas SB occlusion group was characterized with increased noncalcified plaque burden across all three bifurcation segments, the low-density plaque burden was significantly higher in proximal MV (6.6 [IQR 2.8–10.3] vs 3.8 [IQR 1.6–7.7]%, *p* = 0.013) and SB segments (7.3 [IQR 4.9–19.2] vs 6.1 [IQR 2.6–9.5]%, *p* = 0.016) among lesions with versus without SB occlusion (Fig. 4).

### Diagnostic performance of quantitative plaque parameters

**Fig. 5:**
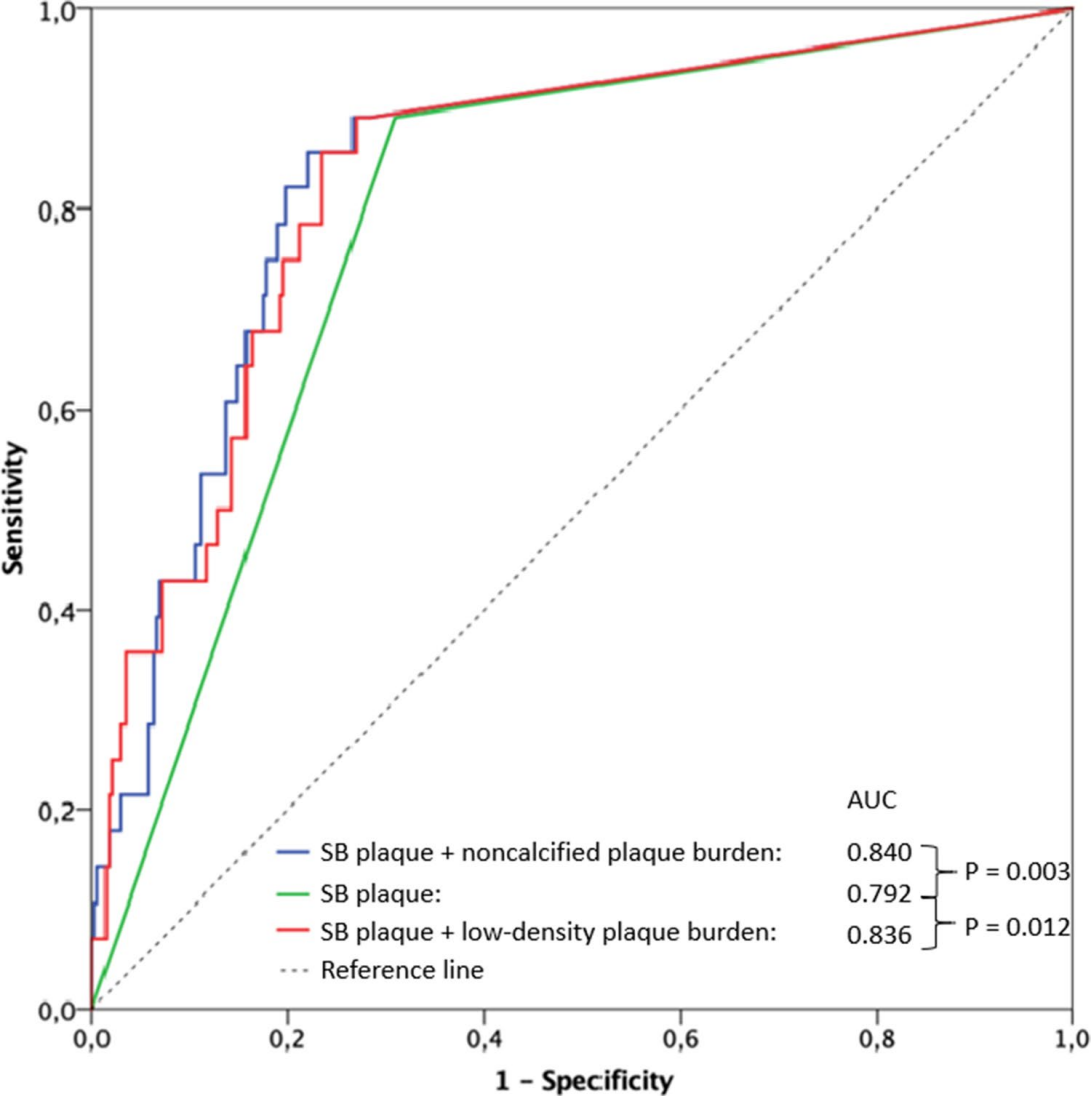
Receiver operative characteristic curves for predicting side branch (SB) occlusion based on visual plaque identification in the SB and addition of noncalcified or low-density plaque burdens

The presence of visually identified plaque in the SB segment, but not in the proximal and distal MV segments, was the only qualitative parameter that predicted SB occlusion with an area under the ROC curve (AUC) of 0.792 (Table 4). Among quantitative plaque parameters calculated for the SB segment, only the addition of noncalcified plaque burden (AUC 0.840, 95% CI: 0.764–0.915, *p* = 0.003) and low-density plaque burden (AUC 0.836, 95% CI: 0.760–0.912, *p* = 0.012) yielded significant improvements in predictive accuracy of visually identified SB plaque presence (Fig. 5).

**Table 4:** Prediction of side-branch occlusion by visually identified coronary plaque in respective bifurcation segments

	Area under the curve	95% confidence interval	Sensitivity (%)	Specificity (%)	*p* value
Proximal main vessel plaque	0.547	0.443–0.650	96%	13%	0.412
Distal main vessel plaque	0.562	0.462–0.661	99%	12%	0.278
Side branch plaque	0.792	0.719–0.867	89%	69%	< 0.001

The presence of coronary plaque in the SB segment had 89% sensitivity and 69% specificity for the prediction of SB occlusion. Using ROC curve analysis, optimal cut-offs for non-calcified plaque burden and low-density plaque burden were > 33.6% (86% sensitivity and 78% specificity) and > 0.9% (89% sensitivity and 73% specificity), respectively.

## Discussion

In this study, we investigated quantitative CT plaque characteristics associated with SB occlusion in patients undergoing coronary bifurcation intervention with initial stenting of the MV. Our main findings are that (1) lesions with SB occlusion have higher total plaque burden resulting from increased noncalcified—but not calcified—plaque deposits when compared to lesions without SB occlusion, and (2) the addition of quantitative noncalcified plaque features to the visual identification of SB plaque significantly improves the prediction of SB occlusion. Notably, the findings from this study may be considered widely generalizable, given the high number of enrolled patients with bifurcations lesions, and semi-automated CTA plaque analysis that has been validated in numerous clinical studies [19, 25–27].

Till now, intravascular imaging allowed to determine two major mechanisms of SB occlusion, mainly the carina and plaque shifts [6–8]. Specifically, it was demonstrated that carina shift results in anatomical SB compromise that might further progress into hemodynamically significant occlusion following the superimposition of coronary plaque [28–30]. Plaque burden within the MV, especially located in its proximal segment, was repeatedly identified as a major predictor for functional SB compromise using different imaging modalities [2, 8, 30, 31]. Moreover, lipid plaques detected with optical coherence tomography were indicated as more prone to undergo shift towards SB during stent implantation [17]. In this context, our results extend prior invasive intravascular observations regarding the impact of plaque morphology on SB occlusion, and add support to the role of non-invasive coronary CTA for planning PCI in bifurcation lesions [8, 17]. Indeed, we demonstrate that higher plaque burden within MV and SB segments of bifurcation lesions is associated with SB compromise following stent implantation. Of note, SB occlusion was primarily driven by increased deposition of “soft” noncalcified plaque. The relevance of low-density plaque located in proximal MV and SB bifurcation segments for occurrence of SB compromise has been recently recognized by Lee et al. [32] In this paper, the authors applied fixed thresholds for quantification of coronary plaque on CTA images acquired with two generations of different vendors’ CT scanners and introduced a semi-quantitatively assessed score to predict SB occlusion with an AUC of 0.749 [32]. Interestingly, quantitative parameters of calcified plaque, believed to be a more stable atherosclerotic component, did not differ across MV segments in our studied groups and were even higher in SB segments of the non-occluded group. Whereas such findings are contradictory with the results of Lee et al., they stand in line with prior intravascular investigations [17, 32]. Importantly, contrary to Lee et al., we used automatically generated scan-specific thresholds for both calcified and noncalcified plaques so as to limit potential bias resulting from discrepancies in acquisition protocols [33]. Our approach was previously validated against intravascular ultrasound; therefore, increasing the reliability of our results [16].

In line with prior studies, we identified the presence of coronary plaque in SB as a robust predictor of its occlusion [2, 15, 31–34]. In our recent paper on CTA-derived RESOLVE score, diameter stenosis of SB before MV stenting and pre-procedural diameter stenosis of bifurcation core achieved the highest diagnostic accuracies for predicting SB occlusion (AUC of 0.720 and 0.725, respectively) [4]. The current analysis demonstrated even higher diagnostic accuracy (AUC of 0.792) for the SB plaque that was visually assessed within the first 5 mm from the SB ostium. Importantly, the diagnostic accuracy of the presence of SB plaque was significantly improved by addition of noncalcified plaque burden (AUC of 0.840). It has been hypothesized that after SB compromise resulting from the shifted carina, only a limited deposit of plaque is necessary to develop hemodynamically significant SB occlusion [30]. We believe that noncalcified or “soft” plaque (contrary to more stable calcium deposits) might be particularly prone for mechanical impairment and subsequent dislodgement with a potential for distal embolization as the consequence of stent implantation across SB ostium. In this regard, the present findings not only reinforce the potential mechanism of SB occlusion, but also emphasize the clinical relevance of CTA-derived noncalcified plaque as a valuable marker for peri-procedural complications in coronary bifurcation interventions [15, 32].

Identification of noncalcified plaque within SB on coronary CTA might be applied for personalized approach in coronary bifurcation interventions. One approach could employ different pre-emptive measures such as shifting from provisional technique to 2-stent strategy with initial stenting of the SB accompanied by stronger antiplatelet (e.g. ticagrelor or prasugrel) and/or antithrombotic therapy during PCI (e.g., use of heparin and/or glycoprotein IIb/IIIa receptor inhibitors under routine activated clotting time guidance). Alternatively, the PCI procedure could be deferred to start intensive statin therapy for plaque stabilization. Finally, referral for coronary artery bypass grafting rather than PCI could be considered in cases with the highest risk for SB occlusion encompassing large myocardial territories.

Our study had several limitations. First, it was a single-center, retrospective and observational study; therefore, we did not account for the influence of stent size or stent implantation pressure on SB occlusion. However, provided that our population was restricted to patients undergoing initial MV stenting with provisional SB treatment, the variability of conceivable interventional techniques was limited, and thus should only marginally affect the rate of SB compromise. Second, the spatial resolution of CT scanners limited our ability to separate the bifurcation core for quantitative plaque analysis. Finally, a relatively low number of SB occlusions might limit statistical power for the detection of differences between studied groups. Nevertheless, our study represents the largest CT database on bifurcation lesions so far, and the percentage of SB occlusions in our report is in accordance with the frequency SB compromise from the large angiographic cohort.[15]

In conclusion, both the total amount of coronary plaque as well as noncalcified plaque is significantly higher in lesions complicated with SB occlusion after MV stenting. The addition of quantitative noncalcified plaque burden to the visually identified SB plaque improves the prediction of SB occlusion. Evaluation of quantitative plaque features derived from non-invasive CTA might help identify lesions at higher risk of SB occlusion following coronary bifurcation interventions.

## Availability of data and material

The data will be available from the corresponding author upon reasonable request.
